# Realist analysis of whether emergency departments with primary care services generate ‘provider-induced demand’

**DOI:** 10.1186/s12873-022-00709-2

**Published:** 2022-09-06

**Authors:** I. J. McFadzean, M. Edwards, F. Davies, A. Cooper, D. Price, A. Carson-Stevens, J. Dale, T. Hughes, A. Porter, B. Harrington, B. Evans, N. Siriwardena, P. Anderson, A. Edwards

**Affiliations:** 1 Division of Population Medicine, Cardiff University School of Medicine, Cardiff, Wales; 2 PRIME Centre Wales, Cardiff University School of Medicine, Cardiff, Wales; 3 Unit of Academic Primary Care, Warwick Medical School, University of Warwick, Warwick, UK; 4 John Radcliff Hospital, Oxford, UK; 5 Swansea University Medical School, Swansea University, Swansea, Wales; 6 School of Health and Social Care, University of Lincoln, Lincoln, UK

**Keywords:** Provider- induced demand, Realist evaluation, Emergency department, Primary care services, Service delivery, Capacity

## Abstract

**Background:**

It is not known whether emergency departments (EDs) with primary care services influence demand for non-urgent care (‘provider-induced demand’). We proposed that distinct primary care services in EDs encourages primary care demand, whereas primary care integrated within EDs may be less likely to cause additional demand. We aimed to explore this and explain contexts (C), mechanisms (M) and outcomes (O) influencing demand.

**Methods:**

We used realist evaluation methodology and observed ED service delivery. Twenty-four patients and 106 staff members (including Clinical Directors and General Practitioners) were interviewed at 13 EDs in England and Wales (240 hours of observations across 30 days). Field notes from observations and interviews were analysed by creating ‘CMO’ configurations to develop and refine theories relating to drivers of demand.

**Results:**

EDs with distinct primary care services were perceived to attract demand for primary care because services were visible, known or enabled direct access to health care services. Other influencing factors included patients’ experiences of accessing primary care, community care capacity, service design and population characteristics.

**Conclusions:**

Patient, local-system and wider-system factors can contribute to additional demand at EDs that include primary care services. Our findings can inform service providers and policymakers in developing strategies to limit the effect of potential influences on additional demand when demand exceeds capacity.

**Supplementary Information:**

The online version contains supplementary material available at 10.1186/s12873-022-00709-2.

## Background

Primary care services are implemented within/alongside Emergency Departments (EDs) to support increasing demand for urgent care, but it is not clear whether this unintentionally leads to further demand for non-urgent care within these settings [1, 2]. ‘Provider-induced demand’ (or ‘supplier-induced demand’) describes when healthcare providers/suppliers create services to attract footfall, funding or income, and patients influence demand with attendance [3]. Provider-induced demand in primary healthcare involves many variables, for example, geographic availability of GPs and growth in service capacity [4]. Ramlakhan et al. describes provider-induced demand here as where healthcare providers generate ‘health-seeking behaviour’ and attendance, in circumstances when barriers, such as access, are removed [5]. Thus, when healthcare services are free, and there is a choice of services for non-emergency conditions, patients may attend newly introduced primary care service within EDs instead of attending primary care elsewhere. This may reflect reduced capacity in community primary care/local urgent care and the increase ED demand may exceed capacity within new services and EDs potentially leading to overcrowding [6].

Patient related factors influencing ED attendance includes difficulties in accessing appointments, dissatisfaction with community primary care services [7], or poor perceptions of the quality of care [8]. Therefore, EDs attendance may be viewed as necessary and a demand-led not provider-induced, feature of service provision. Local system factors influencing demand for primary care at EDs include poor integration of in/out-of-hours primary care, ineffective referral pathways, or increased publicity about new services [2, 6]. Wider system factors influencing demand include national-level policy, strategic and operational delivery of regional services, and access to diagnostic investigations and treatments in EDs [7].

There is mixed U.K evidence regarding the factors that influence ED attendances when primary care services are available [9–14] with increased attendances when primary care services are provided alongside minor injuries units but not with EDs [15–17]. However, evidence shows that integrating primary care practitioners in EDs in European services increases demand [18–20]. Primary care services in emergency departments vary in form and function; some are ‘integrated’ inside the ED, and so less visible while others are ‘distinct’ and more visible to patients because they are separate to the ED. [20, 21]

The aim of this paper is to use a realist approach [22], (Additional file 1) to explore contexts and mechanisms that influence the outcome – i.e., demand for urgent care in emergency departments that include primary care services. Context is defined as pre-existing factors that influence the success or failure of different interventions or programmes. Mechanism details the characteristics of the intervention and people’s reaction to it, or how it influences their reasoning. Outcome is the intended and unintended result of the intervention because of a mechanism operating within a context [22].

We sought evidence to support or refine an initial theory about provider-induced demand within a rapid realist review, exploring the impact of general practitioners working in or alongside emergency departments [1], and to identify new theories.

**Table:** 

**Theory: If patients with primary care problems present to emergency departments (C) and are streamed to integrated primary care services, without awareness or choice (M), there is no provider induced demand (O). However, distinct urgent primary care services may offer convenient access to primary care (M), resulting in provider induced demand (O).**	

## Methods

### Study design

Realist evaluation is a theory-driven approach which considers what is working, for whom, under which circumstances, and how [23]. It is used within healthcare evaluation because it uncovers a deeper understanding of the issues present and potential solutions to mitigate them [24, 25].

The findings reported in this paper are part of a mixed-methods realist evaluation: ‘Evaluating effectiveness, safety, patient experience and system implications of different models of using GPs in or alongside Emergency Departments’ (HS&DR Project 15/145/04) to examine changes in service delivery [26]. We will report results from qualitative data collection, describing the opinions of staff and patients. Further quantitative analysis exploring changing attending rates at EDs with GP-ED models is ongoing and will be reported elsewhere.

### Generation of the study sample

In 2017, we distributed a survey to clinical directors (CDs) of all type 1 EDs (Consultant – led departments, open 24 hours with full resuscitation facilities) in England (*n* = 171) and Wales (*n* = 13). We received 77 responses and chose a sample of 30 EDs with different primary care models as seen in Table 1 [20] to conduct follow up qualitative interviews with CDs [26]; 21 EDs were included in follow up interviews. From these, 13 case study site were purposely selected based on the criteria listed (below) considering the types of primary care models identified in our taxonomy of primary care services in EDs (Table 1) [19]. The 10 EDs included in this paper had GPs and other primary care clinicians working within them, three as ‘inside-integrated’ models (Hospitals, 3, 8 and 14,) four as ‘Inside-parallel’ (Hospitals 4, 6, 7, 9) and three as ‘outside-onsite’ (Hospitals 10, 11,13) [20]. The three EDs not included did not have GPs/primary care clinicians working within them.

**Table 1:** Primary care models [20]

**Inside – integrated services (I-I)**	Primary care services fully integrated within EDs. Staff review primary and emergency care patients (*n* = 3). These **were not visible** to patients/patients generally unaware of GPs working in EDs (Hospitals 3, 9, 14).
**Inside-parallel services (I-P)**	Separate (distinct) primary care service within ED for patients with primary care type problems (*n* = 4), **were not visible** (Hospitals 4, 8), **or visible but separate** and patients were streamed by ED/111 (telephone service): patients were unaware of them (Hospital 7), or services accessible from ED that the public were aware of (Hospital 6).
**Outside-onsite sites (O-O)**	Separate (distinct) primary care services on-site (*n* = 3) **were visible**, offering walk-in services that the public were aware of (Hospitals 10,11). Or primary care services within different part of the hospital and patients streamed from ED/111 (Hospital 13).

All interviews were audio-recorded and transcribed verbatim.

### Criteria for case study site selection

- Primary care service in ED since 2010.
- Variation in service model – delivering **distinct** primary care services, inside or outside EDs or a primary care service **integrated** with the ED.
- Spread across England and Wales.
- Variety of contexts – including rural/urban locations, small/large hospitals, higher/ lower attendances.
- Variation in streaming method – who, streaming criteria and guidance.
- Variation in the physical layout of ED.
- Variation in relationship with the GP out-of-hours services.

### Data collection (see Additional file 2 for interview guide)

#### Pre-visit interviews with CDs

Telephone or in-person interviews took place between February 2018 and March 2019 (~ 60 minutes) [27]. During these interviews CDs were asked about service operation, perceived successes, and challenges in providing and delivering services and how their experiences related to our theories. Ethical approval for the survey and follow-up interviews with CDs was given by (anonymised) Ethics Committee (ref: 17/45).

#### Observations and interviews with staff at study sites

Researchers (ME and AC) undertook 2–3-day visits to study sites between February 2018 - April 2019. Visits took place when primary care clinicians were present within ED over approximately 8 hours on average. Patients were observed throughout their care journey from arriving at the reception, triage and streaming assessments and formal and short informal interviews were conducted with nurses and other clinicians. The realist teacher-learner interview technique was used to present initial theories and explore how different mechanisms in different contexts may result in intended and unintended outcomes [23].

#### Patient interviews

As described by Price et al. [27] we also carried out semi- structured interviews with 24 patients/carers who visited EDs for one of six conditions (chest pain, cough and breathlessness, abdominal pain, back pain, headache and fever in a child under 10 years old). These were considered by stakeholders (academics, primary care and ED clinicians and patient and public contributors) as conditions that could be managed by primary care clinicians or ED clinicians, and were identified using literature on ambulatory care sensitive conditions [22, 28–35] and our stakeholder group [20]. Patients were purposively sampled and contacted via post within 12 weeks of their visit to EDs or by members of NHS staff during site visits, to inform about eligibility to take part in the study and request their consent for interviews. Interviews were conducted by telephone by ME between February 2018 and March 2019 (over ~ 20 minutes). Despite experiencing difficulties with recruitment and access to patient participants [27], the purposive sample included adults of different ages, parents of children, and people with different conditions from the three primary care models (Table 1).

### Data analysis

We analysed data from observations and telephone interviews with CDs and case study visits. We used a realist approach, generating ‘context-mechanism-outcome’ (CMO) configurations [35] from the data. We did this by identifying mechanisms that relate to influences on demand and the contextual factors that influence those mechanisms. We then mapped CMO configurations against different primary care service models [20] and factors perceived to influence demand based on Pawson’s theory-building processes (juxtaposition, reconciliation, adjudication, and consolidation) [22]. We incorporated expert knowledge of primary and emergency care academics and public contributors in theory refinement and development by discussing early findings within the study team and co-investigators and refined analysis based on feedback.

### Stakeholder engagement

We presented our theories at a stakeholder workshop [20] with 56 attendees including ED staff, GPs, service managers, policymakers, patients, and public contributors. They provided feedback and suggested additional contexts and mechanisms for consideration. In the final stage of the analysis, we identified relevant middle range theories which we used as a lens to interpret our results [22]. These informed the development of our programme theory which summarises the findings of this work.

### Interpreting results through a theoretical lens – using middle range theory and generating a programme theory

We used Richardson’s analysis of supply and demand in health care [36] as a middle range theory to interpret the findings and theories emerging from our study [22]. We aimed to integrate these theories as a ‘Programme Theory’ to explain and summarise why using primary care clinicians in or alongside EDs may or may not lead to provider-induced demand, for whom, and in what specific circumstances. A programme theory is an overall high-level theory summarising how the intervention works, developed using the theories refined from the data [22].

### Patient and public involvement

Patients and public members were involved in the study design [26] and as co-applicants in the funded study in line with best practice [36], discussing their experience as NHS patients to contribute to this research. They advised on data collection tools and patient recruitment when the team experienced difficulties [27]. They supported involvement of public and patient contributors to the stakeholder event and were involved in discussing draft data and paper preparation [37].

## Results

We used qualitative data from: interviews with patients, (*n* = 24), ED doctors, ED Nurses and GPs (*n* = 106), field notes of observations by two researchers from the 10 study sites, and input from stakeholders involved in guiding the selection of patient groups and discussing influences on demand (*n* = 56). Based on our findings, we noted three distinct levels (patient, local systems and wider (regional or national) systems) in which we could describe influences on demand (Table 2).

**Table 2:** Levels and themes contributing to theories

Level	Themes	Quotes	Theories
**1. Patient** Patients attend EDs based on knowledge of their medical conditions or due to convenience/ preference.	**A. Experience and assessment of condition:** Clinical Directors (CDs) reported patients are “correct” in choosing which services to attend (Hospitals 3, 4, 6, 11) and stated that those who attend are sicker than patients who attend community primary care (Hospitals 3, 4, 18). A patient in one hospital reported that she knew when to take her child to the ED because of past experience (hospital 3)	“[We’ve been in] hospital once a month…admitted through the GP or knowing the signs and going to A&E to get him checked out.” **Parent of child seen by GP, Hospital 3 (inside-integrated model).**	**A**. Patients with persistent health problems who have previously accessed EDs (C), or with potentially serious symptoms, have good knowledge and seek emergency care EDs (M) and judge that it is appropriate to attend EDs rather than community primary care services (O).
	**B. Convenience (location or time) & C. Preferences (including second opinions)** Patient’s access EDs with separate (distinct) primary care services in EDs if they are geographically convenient or at convenient times. Reception staff revealed that services can be accessed all day (Hospitals 6, 10, 13). Clinicians (Hospital 6) stated that patients attend EDs in the morning, despite having afternoon appointments GPs, because they are at the hospital for other appointments or when accompanying relatives e.g., a patient attended for an ultrasound in the hospital and had a foot problem, so attended the ED too.	“[Patient had appointment with GP] but thinks ‘if I go to the hospital now, I’ll be done by lunch time’. And [within] consultations say “excellent, that means I don’t have to go to my GP”. **CD, Hospital 6, (inside-parallel model).**	**B.** Patients with difficulty accessing GP appointments in an acceptable timeframe (C) they may believe that EDs are the best place to attend for urgent care (M) and attend EDs with a distinct primary care service for emergency or primary care services (O).
			**C.** Patients who live /work closer to EDs (especially those with a visible and distinct service) are more likely to attend there with a primary care problem (C) because of convenient local access where people expect to be seen more quickly than in community primary care or for a second opinion (M), generating additional demand for primary care at the ED (O)
**2. Local systems** Whether patients can access care and referral pathways or new buildings and publicity.	**A. Access to community primary care/hospital services** Some local primary care services were perceived as inaccessible (Hospitals 4, 5, 14) and the 111 service directed patients to EDs, which increased demand for primary care services in the ED. Patients unable to access timely GP appointments attended EDs either, with the intention of accessing emergency care (Hospitals 13, 16), or to access an inside-parallell (distinct) primary care services at an ED (Hospital 6). These influences increased demand for primary care services and overall, ED service workload.	“I was breathless for, well, days beforehand and **couldn’t get an appointment at the doctors** and I thought, well, I’ll just go up to the **walk-in centre at the hospital**.” Patient seen by ED clinician **(Hospital 6 – inside-parallel model)**	**A.** When patinets perceive that they are not able to access local primary care services (C), they may choose to access an ED with a distinct primary care service or they contact 111 service and are advised to seek urgent care at an ED (M) thus generating additional demand in the ED (O).
	**B. Urgent care referral pathways** Some patients were advised to attend an ED by community primary care services due to their insufficient capacity to see urgent presentations, 111 services also referred primary care patients to an inside- integeated ED when they had conditions that would be more suitably treated within community primary care.	“[Patients say] “we phoned 111 and they said go to A&E” … 111 is not a re-direction service… it’s a misdirection service…”, **CD, Hospital 14 (inside-integrated model)**	B. When primary care services have to refer patients to an ED because they have no capacity to see urgent care patients or patients are inappropriately assessed by the 111 service as having a problem that could be seen in the ED (C) patients that could otherwise be seen in community primary care are sent to an ED (M) thus generating additional primary care demand in the ED (O).
	**C. Service improvements & publicity** Service developments such as new buildings or renovations to add a primary care services to an ED were seen by CDs to potentially influence additional demand (hospital 3 and 6), and was reported to be evident where there is a distinct service that patients can walk in to (hospital 6) Publicity, and increased public knowledge about services also predisposed patients from wider areas to attend (Hospital 6).	“When we opened this building, our attendance rate went up 30%... We started to see whole populations coming to us which never came before.... When you put the service and make it available, it generates work.” **ED consultant, Hospital 6, inside-parallel model)**	**C.** Service developments involving new or renovated buildings (C), media publicity and increased public awareness, may lead to patients from local and further areas choosing to attend the service (M) creating additional demand for emergency and primary care (O).
**3. Wider system (regional / national influences**)	**A. Population characteristics** Populations of patients were sometimes viewed by staff as able to judge which conditions were “appropriate” to present to the ED or considered “stoic” in terms of their health-seeking behaviour. In some areas, populations were characterised as having large numbers of temporary residents, such as visitors, tourists, and transient workers who, due to unfamiliarity with services, choose to attend EDs if unwell. In diverse cities in which people have recently arrived in the UK, or with large student populations, different cultural perceptions of accessing healthcare, or not being registered with a GP were factors considered to make it more likely to seek primary care at EDs.	“We see minor injury and fractures, and it’s linked with the rural population, in that a high proportion do have fractures or true injuries”. **CD, Hospital 9, (inside-integrated model)**	**A.** In areas where people are not registered with a GP, have different cultural perceptions about how to access health care, or are unfamiliar with local services (C), patients may attend EDs for primary health care needs (M), and generate demand for primary care at the ED (O). Similarly, in areas with less diversity, or rural areas (C), attendances were perceived to be associated with minor injuries and fractures (C), and staff report that people tend not attend EDs with primary care problems (M) ensuring less demand for primary care in EDs (O).
	**B. Service improvements & unintended consequences:** We noted a regional 111 directive of advising patients to attend EDs with the shortest waiting times.	“Somebody who lived in [elsewhere] was told [by 111] to come here, bypassing the ED at [other hospital] to come to us…all his follow-up will now be under us” **Nurse, Hospital 6, (inside-parallel model)**	**B.** Service improvements that focus on waiting times (C) may lead to patients being referred to other EDs (M) which creates extra demand and workload at more efficient hospitals (O).

### Interpreting results through a theoretical lens

Economic theory of supply and demand typically focuses on suppliers and consumers. However, economic analysis of health care markets, includes patient, provider (clinicians), agencies financing health services and the government/regulator in the UK/ NHS. According to Richardson [36] a satisfactory theory of provider-induced demand must answer:

(i). Why patients allow their preferences to be changed/manipulated.
(ii). What motivates clinicians and, why they fail to maximise demand shifts to achieve objectives including increasing income or leisure, care quality or professional satisfaction [4].

In the UK, healthcare costs are not directly incurred by patients during health care encounters, so it can be argued that where typically provider-induced demand focus is on clinicians maximising benefit through supply, but within the UK the patient is the agent seeking to maximise benefit (e.g., convenience, quality of care) from an individual encounter (through demand). Thus, questions might be reframed as:

(i) Why do service providers allow appropriate treatment provision to be manipulated?
(ii) What motivates patients, and why might they fail to maximise demand shift and leave unexploited an opportunity to increase their benefit?

There is evidence of “supplier-induced demand” in healthcare in the context of a complex interaction of many variables. For example, an Australian review noted research reporting “close relationships between the geographic availability of GPs and the use of services” and that “over time there has been an almost perfect correlation between growth of general practitioners and the use of their services” [4]. The additional capacity could be used for greater time with patients, better quality care, or may enhance professional satisfaction.

Our findings suggest that patients have motivations that reflect prior experiences, judge severity of their conditions, and seek help appropriately, but sometimes for convenience or satisfaction. Some patients may have their needs met by arriving at the ED and this can contribute to additional demand. However, they may also be directed away from the ED to community primary care services of assessment or demand for non-urgent care is too great at the ED. At local system levels, service providers can unintentionally enable access to urgent care to be manipulated. Supply issues are complex and reduced capacity of in-hours/out-of-hours primary care, and provision of primary care services within ED services can encourage patients to attend EDs, thus directly increasing demand. Referral pathways (deliberate and unintentional) have been seen to reinforce this shifting the balance of demand towards the ED. Wider system level factors such as population characteristics, including patients not registered with GPs for example: tourists, can lead to increased demand for urgent and primary care in the ED. Whilst some services may introduce walk-in centres to address these factors, these become overwhelmed if patients are not educated and signposted to ‘appropriately’ access primary care (in- and out-of-hours) and EDs.

### Programme theory

**Fig. 1:**
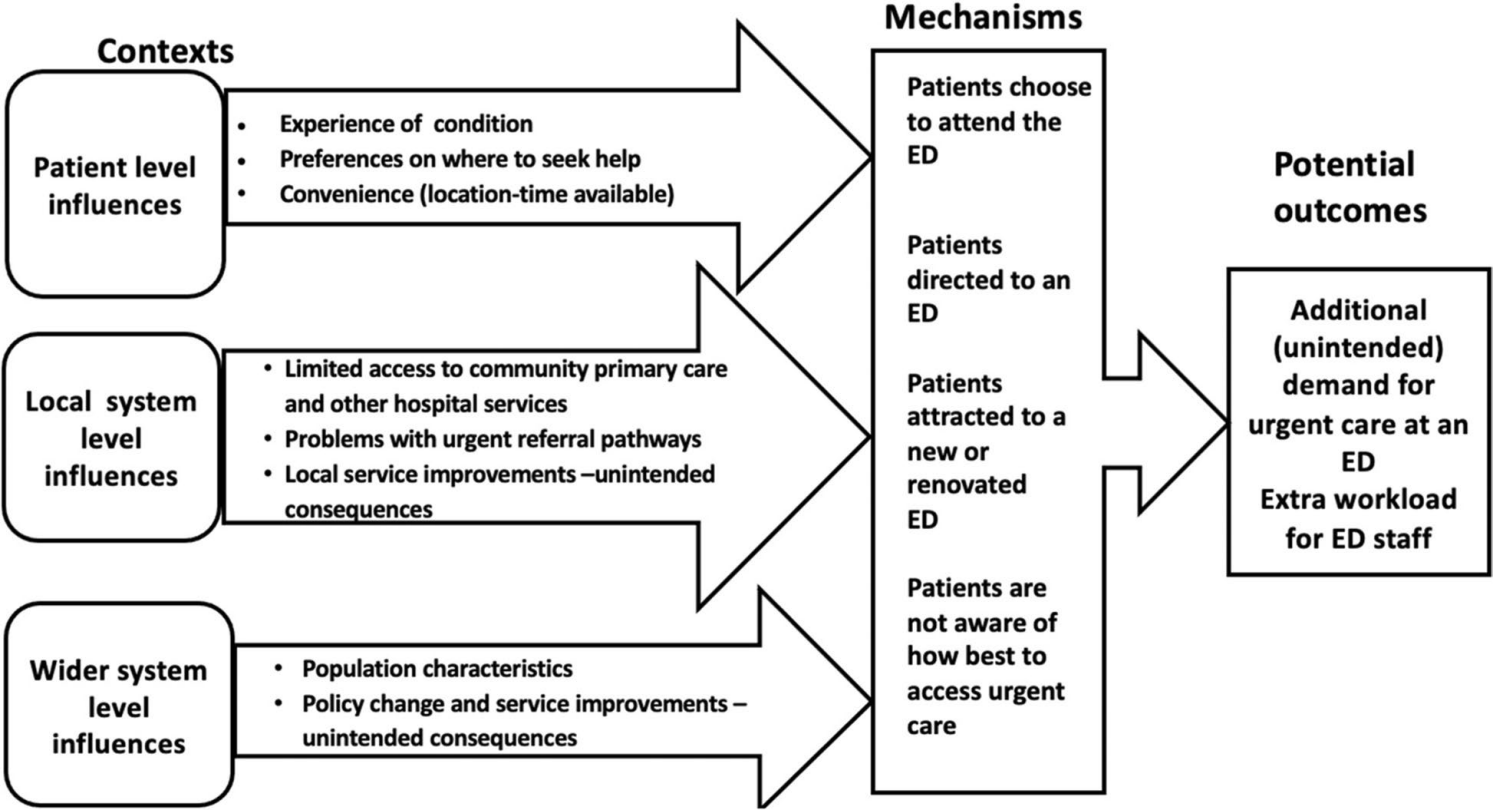
Programme theory

We integrated our refined theories as a Programme Theory [22] which reflects patient motivations for accessing EDs and how local and wider system level factors influence service provision and unintentional increased demand (Fig. 1).

## Discussion

### Principal findings

The results in Table 2 indicate that inside-parallel models and outside-onsite models (with distinct services) [20] are perceived by staff to receive additional demand from 111 services and community primary care services appropriately and inappropriately referring urgent primary care patients to them and are more likely to attract additional demand from patients, because they are more visible to patients, widely known about, or sometimes enable direct access to urgent care. Therefore, our initial theory that ‘distinct’ services are more likely and integrated services are less likely to generate provider-induced demand appears to be supported [4].

Provider-induced demand was reported by clinicians to be more likely in circumstances relating to local or wider systems, particularly for primary health care, especially when it had little capacity to accommodate urgent care requests, or among population groups such as those not registered with a GP or new to the area and less familiar with pathways and ways to access care for needs. In this context patients naturally responded to lack of provision in one sector and accessing care where it was more available (EDs).

### Strengths and weaknesses

We used rigorous methodology to carry out a realist study [24], including theory generation and refinement based on input from a range of stakeholders from academia, clinical practice and patient and public contributors. Our findings on patient experiences are based on conditions that were identified as urgent care conditions that patients might present with and are seen by a primary care or ED clinician. However, we acknowledge that our findings may not be generalisable to all patient groups that attend an ED for urgent care.

Our findings and theory refinements and generation reported here are based on reports by staff and patients; in a further report we plan to analyse time-series data on ED attendances at the study sites to look for evidence of increases in demand after these primary care services were implemented.

### Context of other literature

Our findings reflect other literature on influences on demand where patients have reported dissatisfaction with and poor access to community primary care services [7, 8], and local system [7] and wider system [6] influences. Concerns about provider-induced demand identified elsewhere [2, 7] appear justified based on our findings, especially relating to reported associations between new buildings (and publicity) and local service improvements contributing to additional demand.

### Implications for policy and practice

We suggest specific ways that provider-induced demand can be managed at local and wider system levels (see Table 3 below). More capacity is needed in community primary care services to support patient access for urgent primary care needs, and pathways and capacity need to be established to safely redirect patients from the ED to other hospital and community-based services. The urgent ‘111’ health advice line and other services need to consider ED capacity and implications of directing patients to services further away. Publicity about new services and service improvements must ensure that patients are informed about which services are most appropriate for urgent care needs. Furthermore, education and information are needed to help modify the behaviour of the few patients who choose to use EDs for their primary and urgent care needs due to convenience or a lack of awareness of how to access community services. Whilst urgent services are necessary and useful for some population groups that typically do not register or are less familiar with community primary care services, education and information may be useful to support them to access primary, urgent and emergency care from community services.

**Table 3:** Summary recommendations for policy and practice

Access	Local primary care services need greater capacity.ED pathways must direct patients to other hospital services (such as ambulatory care) and community primary care services
Appropriate referrals	111 services must consider capacity for primary care at EDs and refer to community primary care services, referring appropriate patients to primary care services in EDs only with appointments..
Publicity	Media about service developments must include education about when access to EDs /primary care services is appropriate.
Waiting times	Limits are needed on the number of patients who are referred to EDs from areas that hospitals are not commissioned to treat.
Education	Information and support should be provided to patients in specific population groups (for example tourists) to support them to register with community primary care services

We have previously identified [36] relatively weak levers with which to balance workforce supply and patient demand and explicit workforce planning must be undertaken. This may be undertaken at local level (signposting of services, referral pathways that do not perversely incentivise inefficiency), but may be more meaningful at “wider system” level (commissioning policy to place capacity where it is most needed, health education programmes to address cultural perceptions of how, when, and where to access services).

Services requires reform to achieve allocative efficiency and the use of evidence-based approaches to achieve “right care for the right patient at the right time (and place)”[4, 39]. Within the present framework, the policy challenge is to determine ways of delivering more cost-effective services while simultaneously achieving equity objectives and maintaining patient autonomy. In summary, it may be necessary for regional authorities or governments to intervene in precisely the way in which economists generally eschew.

### Further research

Our findings can inform research to further understand the socio-demographic factors that influence why patients attend primary care services at EDs, and to examine the extent of demand changes with different types of service provision. If there is provider-induced demand, quantification of overall benefits is needed to assess whether it may still be acceptable, if safe quality care is provided and if it makes ED workload pressures manageable. Similarly, evaluation is required at the level of the health economy into whether the cost of implementing primary care services at EDs delivers most benefit at that site compared to improving primary care and urgent care services outside the ED. Evaluations are also needed of improved urgent care pathways which seek to ensure that referrals are appropriate, and patients are streamed suitably – whether within EDs or primary care services.

## Conclusion

Our findings suggest that primary care services implemented within/alongside EDs can encourage additional demand at EDs, with both visibility of direct access for patients and local urgent care referral pathways contributing to this. We have described a range of patient, local-system and wider-system level factors that contribute to additional demand. Our findings can inform providers and regional policymakers to develop strategies to mitigate the potential effects of these influences on demand.

## Supplementary Information

**Additional file 1.**

**Additional file 2.** Interview guide for telephone interviews with clinical leads

## Data Availability

Data generated or analysed during this study are included in this published article [and its Supplementary information files], other information is available from the corresponding author on reasonable request.
