# Supplementary material for: Realist analysis of whether emergency departments with primary care services generate ‘provider-induced demand’
Source: BMC Emerg Med. 2022 Sep 6;22:155. doi: 10.1186/s12873-022-00709-2 (PMC9450363; doi:10.1186/s12873-022-00709-2)
Supplement: Supplementary file 2 — Additional file 2. Interview guide for telephone interviews with clinical leads [file 12873_2022_709_MOESM2_ESM.docx]

**Additional file 2 Interview guide for telephone interviews with clinical leads**

**Example from hospital 10**

**Department level – Demand/Flow**

| **Question** |
| --- |
| - In the survey you say that you have GPs working in a separate area next to the ED with a separate entrance, is that right? - How is the place where the GPs work labelled? Is it labelled as an urgent care centre? It is known by the public that there are GPs working there? |
| - So an ED nurse selects patients to be seen by a GP using locally developed inclusion criteria, can you explain more about how that works? - Is there a triage system or streaming system whereby the nurse can stream the patient to a GP or another member of staff? What are the options? Can you explain how the streaming works? - And there is a telephone triage where primary care service can make an appointment, can you explain how that works to me? - And patients can walk in and be seen? - So patients who need urgent care could be seen by a GP, ANP, ENP or Paramedic ACP, is that right? |
| - How does having GPs in your department affect the flow of patients in the ED? - Is there any exchange of patients from the GP area to the ED area and vice versa? |
| - You mentioned a home visiting service provided by advance care paramedics, can you explain more about how that works? |
| - The literature suggests that when people know that there are GPs working in the ED that they are more likely to go to the ED and so there is a provider induced demand. - We think that provider induced demand might occur more where there are separate areas where it is know that GPs work from as opposed to EDs where the GPs are embedded into the ED. Do you think that happens here? |
| - In the survey you say that there is also a GP out of hours service located at the hospital, is this a separate service to the GP service next to the ED (or the same)? - Were they previously separate and have now merged? - You mentioned in the survey something about the contract for OOH being renewed to include a 24/7 GP presence, 24 hour walk in and adjacent to the ED. Can you explain more about that? |

**Department level - Meeting the aim of GP service**

| **Question** |
| --- |
| - The literature suggests that GPs might admit less patients and do less investigations for some patients than a junior doctor for example. We have also found this talking to some other departments? - Are the GPs able to admit patients? - Why do you think that having GPs has not reduced hospital admissions in your department? |
| - You also said that most of the time there are improvements in quality of care given to certain types of cases |
| - You said that the GP service enables better use of available ED resources. Can you explain more about this? |
| - You also said that the GP service is never cost saving, why is this? |

**Department level- Changes in the service**

| **Question** |
| --- |
| - So you said a change of contract helped with setting up the service. So the contract is now held by the acute trust - What were the previous arrangements - previously contracts held separately for OOH and walk in by pseudo private and private providers |
| - You have said that your ED is making a capital bid, what does that involve? |

**Practitioner level questions**

| **Question** |
| --- |
| - You say that in addition to primary care and low acuity type patients - We notice that GPs have access to mainly tests that can be accessed in primary care and can order x-rays. Do patients who need X-rays remain in the urgent care centre or are they passed over to the ED? |
| - Another hospital has told us that the biggest advantages of having GPs working in the ED and not close by is the exchange of patients and that GPs and ED clinicians have the opportunity to seek advice from each other? Does this happen in your department? - Are there any learning experiences for GPs and ED doctors from working in a department with an urgent care centre next to it? |
| - We know from what other hospitals have said that there are problems recruiting GPs with the appropriate skillset to work in the ED. - Do the GPs working in the department there need to have a different skillset from GPs that might work in a local surgery? Might they see more,minor injuries or sicker patients? |
| - Do your junior doctors in the ED get to see patients with primary care problems? - Do you think they get to see enough of these patients? |
| - Have there been any issues with recruiting and staffing with GPs? We know in some areas it has been difficult to recruit GPs to work in the ED and sometimes a specific skillset is needed which can be difficult to meet. |

**Patient safety**

| **Question** |
| --- |
| - Do you think there are any positive or negative safety implications related with having GPs working in the ED? |
| - Some hospitals have reported rare cases of missed diagnoses from GPs seeing patients in the emergency department - are you aware of any such events? Were any changes made as a result to minimise future events? |
| - The literature suggests that having GPs working in the ED mean that the ED doctors can see more seriously unwell patients quicker, is that the case here? |

**Wider system level**

| **Question** |
| --- |
| - Do you think that having GPs in the ED produces local competition for local jobs? - If yes, then does that then make it difficult to for primary care to manage the demand in their service? |
| - Do you have any types of patients that visit the ED with primary care problems that might have problems accessing traditional primary care? For example, tourists, migrants, vulnerable groups |

**ED staff interview guide**

**Introduce the study to the participant**

Thank you for taking part in the interview today. Our aim is to talk to you about you about your role and how the GP-ED model works. We are studying 12 hospitals in England and Wales as part of an NIHR funded project, 9 that have GPs and 3 that do not. We hope to explore what kind of models work in different contexts and what outcomes are achieved. Your interview will form part of our qualitative research evidence and then we will be extracting hospital episode data to look at outcomes such as admissions, use of resources and costs etc.

The interview can last for as long as you are available to speak to me today, please feel free to pause or stop the interview if there is something else you need to deal with. What we talk about during the interview will not be linked to your name as we use ID numbers for all hospitals and staff members that we interview. Please read the patient information and sign the consent form and we can begin the interview when you are ready.

**Introductory questions**

- Staff title/role in the department?
- How do you feel the GP-ED service works here?

| **Themes** | **Questions** |
| --- | --- |
| **Streaming** | - Can you explain how the **streaming/ triage works**here? (prompts seniority of streamer, usual time waiting to triage/streaming, different targets for ED and GPs?, whether obs are taken routinely or not, influenced by workload? Patients referred by local GP?) - Do you have any guidance on specific conditions and where they should be streamed? - We know from the literature that **experience and clinical judgement may affect how guidance is interpreted who is streamed to GPs.** What is your experience of this? - Prompts - changes/adaptions, what may influence which patients are streamed to GPs, are there enough primary care patients to stream to GPs? |
| **Role of the GP** | - What **role do the GPs** adopt in the department?(gatekeeper/traditional/extended/ED clinician) - Prompts – what influences this role? - We have a theory that **GPs may manage patients differently to ED clinicians** (less investigations and admissions)?   For example a patient with abdominal pain, non-specific chest pain or child with a fever  What is your experience of this? What influences this?  Prompts – in what situations? Time of day? Type of patient? Experience of doctor?   - We have a theory that **GPs may manage patients differently when working in an ED setting than they would if they saw the same patient working in the community** - **How about compared to the same patient in OOH?**   Have you any experience of this?  Prompts – abdominal pain/non-specific chest pain/child with a fever  Context – type of patient/experience of doctor/time of day/streamed as a ‘patient with a primary care problem likely to go home’? |
| **Flow/**  **Provider induced demand** | - Does the GP-ED model affect **flow in the department**?   Prompts – 4 hour targets, freeing up ED staff to see the sicker patients quicker, what is it like when there are no GPs or before the GP-ED model  why – less investigations/admissions? Seeing the non-urgent patients?   - There is some suggestion in the literature patients get to know that there are GPs in the ED and use the service as a **convenient access to primary care, which increases the presentation of patients with primary care problems**, have you any experience of that happening here? |
| **Patient experience** | - Does the GP-ED model have an impact on patient experience?   Prompts – less waiting time? Less investigations?   - Frequent attenders that use the service instead of local primary care – do they miss out on long term care? |

| **Team working/**  **learning/**  **integration** | - How do the GPs fit into the ED team?   Prompts – same meetings/governance/social events/informal conversation?   - Are there any positive **learning experiences for GPs and ED doctors** by working alongside each other? - In particular for junior doctors, there are some reports in the literature that they may **miss out on learning opportunities** because the GPs are seeing a certain group of patients but our research suggests that some junior doctors find the GPs a useful resource for senior advice. Have you any thoughts on this? |
| --- | --- |
| **Safety** | - There is very little in the literature about the **safety implications** about GPs working in or alongside EDs.Are you aware of any positive or negative safety implications?(Prompts primary care patients less over investigation?) - Are there any specific conditions that you feel GPs manage well or not so well? - We’re exploring the potential**risk of GPs seeing sicker patients than they usually deal with in practice or conditions outside of their skillset**, do you have any experience of this? - Potentially GPs may be **using investigations that they are less familiar with** interpreting such as XRays – are you aware of any such issues? - For GPs - **Any learning / adaptions to their diagnostic approach** in an ED setting **compared to usual practice** (explore if they are aware of any change in cognitive biases? certain presentations/conditions, more cautious or not, more likely to investigate or not)? - GPs may be streamed low risk patients not felt to require ED level investigation (e.g. chest pain/headache/musculoskeletal injuries) - have you thought about how this may influence your management? - Any **recommendations/training to GPs** about to start working in an ED setting? |
| **Wider system** | - How would you describe the **local GP service**? What is it like for getting appointments? - Have there been any problems **recruiting and retaining** ED staff and/or GPs to work in the ED? - One site reported that the **GPs enjoy the work** and employing GPs in the ED may help retain this workforce in the NHS, any thoughts on this? |
| **Cost-effectiveness** | - There is some evidence that **GPs seeing patients with primary care problems in ED may be cost effective due to the reduced number of investigations and admissions,** have you any thoughts on this? |

**Thank you for taking the time to be interviewed today, your responses are valuable to us understanding how GPs work in the department here. Are there any questions that you have or any other comments that you would like to make?**

**Themes and sub-themes relating to streaming and redirection from clinical lead interviews**

| **1.0** | **Methods of streaming** | **2.0** | **Redirection** |
| --- | --- | --- | --- |
| 1.1 | Streaming at the front door | 2.1 | Redirecting to community |
| 1.2 | Streaming combined with triage | 2.2 | Redirect with an appointment |
| 1.3 | Complex streaming | 2.3 | Stream primary care patients to the urgent care centre to be redirected |
| 1.4 | Streaming children to a clinician | 2.4 | Not feasible to redirect to GPs because of distance |
| 1.5 | Parallel streaming - nurses in ED and UCC | 2.5 | Signposting patients to another service for their ongoing problem |
| 1.6 | Stream to geriatric emergency care |  |  |
| 1.7 | Streaming to slots in ooh GP service |  |  |
| 1.8 | GPs self select patients |  |  |
| 1.9 | Triage only - no streaming |  |  |
| 1.10 | Non-clinical streaming |  |  |
